# Gastrodin attenuates diabetic cardiomyopathy characterized by myocardial fibrosis by inhibiting the KLK8-PAR1 signaling axis

**DOI:** 10.1186/s13020-024-01035-4

**Published:** 2024-11-22

**Authors:** MingShan Zhang, YuFei Zhang, JingGang He, XinRui Wang, YinYin Wang, LinYan Li, Ling Tao, Min Zhang, Xiangchun Shen

**Affiliations:** 1 The State Key Laboratory of Functions and Applications of Medicinal Plants, The Key Laboratory of Endemic and Ethnic Diseases of Ministry of Education), Guizhou Medical University, No.6 Ankang Avenue, Guian New District, Guiyang, 561113 Guizhou China; 2 Department of Physiology, School of Basic Medical Sciences, Guizhou Medical University, Guian New District, Guiyang , 561113 Guizhou China; 3 The Department of Pharmacology of Materia Medica (The High Efficacy Application of Natural Medicinal Resources Engineering Center of Guizhou Province and The high educational key laboratory of Guizhou province for natural medicianl Pharmacology and Druggability), Guian New District, Guiyang, 561113 Guizhou China

**Keywords:** Diabetic cardiomyopathy, Myocardial fibrosis, Gastrodin, KLK8

## Abstract

**Background:**

Diabetic cardiomyopathy (DCM), characterized by myocardial fibrosis, is a major cause of mortality and morbidity in diabetic patients; the inhibition of cardiac fibrosis is a fundamental strategy for treating DCM. Gastrodin (GAS), a compound extracted from *Gastrodia elata* protects against DCM, but the molecular mechanism underlying its antifibrotic effect has not been elucidated.

**Methods:**

In vivo, the effects of GAS were investigated using C57BL/6 mice with DCM, which was induced by administering a high-sugar, high-fat (HSF) diet and streptozotocin (STZ). We assessed the cardiac function in these mice and detected histopathological changes in their hearts and the degree of cardiac fibrosis. In vitro, neonatal rat cardiac fibroblasts (CFs) were transformed into myofibroblasts by exposing them to high glucose combined with high palmitic acid (HG-PA), and CFs were induced by pEX-1 (pGCMV/MCS/EGFP/Neo) plasmid-mediated overexpression of KLK8, which contains the rat KLK8 gene. The KLK8 siRNA was knocked down to study the effects of GAS on CF differentiation, collagen synthesis, and cell migration by specific mechanisms of action of GAS.

**Results:**

GAS attenuated pathological changes in the hearts of DCM mice, rescued impaired cardiac function, and attenuated cardiac fibrosis. Additionally, the results of molecular docking analysis showed that GAS binds to kinin-releasing enzyme-related peptidase 8 (KLK8) to inhibit the increase in protease-activated receptor-1 (PAR-1), thus attenuating myocardial fibrosis. Specifically, GAS attenuated the transformation of neonatal rat CFs to myofibroblasts exposed to HG-PA. Overexpressing KLK8 promoted CF differentiation, collagen synthesis, and cell migration, and KLK8 siRNA attenuated HG-PA-induced CF differentiation, collagen synthesis, and cell migration. Further studies revealed that a PAR-1 antagonist, but not a PAR-2 antagonist, could attenuate CF differentiation, collagen synthesis, and cell migration. Additionally, GAS inhibited KLK8 upregulation and PAR1 activation, thus blocking the differentiation, collagen synthesis, and cell migration of HG-PA-exposed CFs and triggering TGF-β1/Smad3 signaling.

**Conclusion:**

GAS alleviated pathological changes in the hearts of DCM model mice induced by an HSF diet combined with STZ. KLK8 mediated HG-PA-induced differentiation, collagen synthesis, and the migration of CFs. GAS attenuated the differentiation, collagen synthesis, and migration of CFs by inhibiting the KLK8-PAR1 signaling axis, a process in which TGF-β1 and Smad3 are involved.

**Graphical Abstract:**

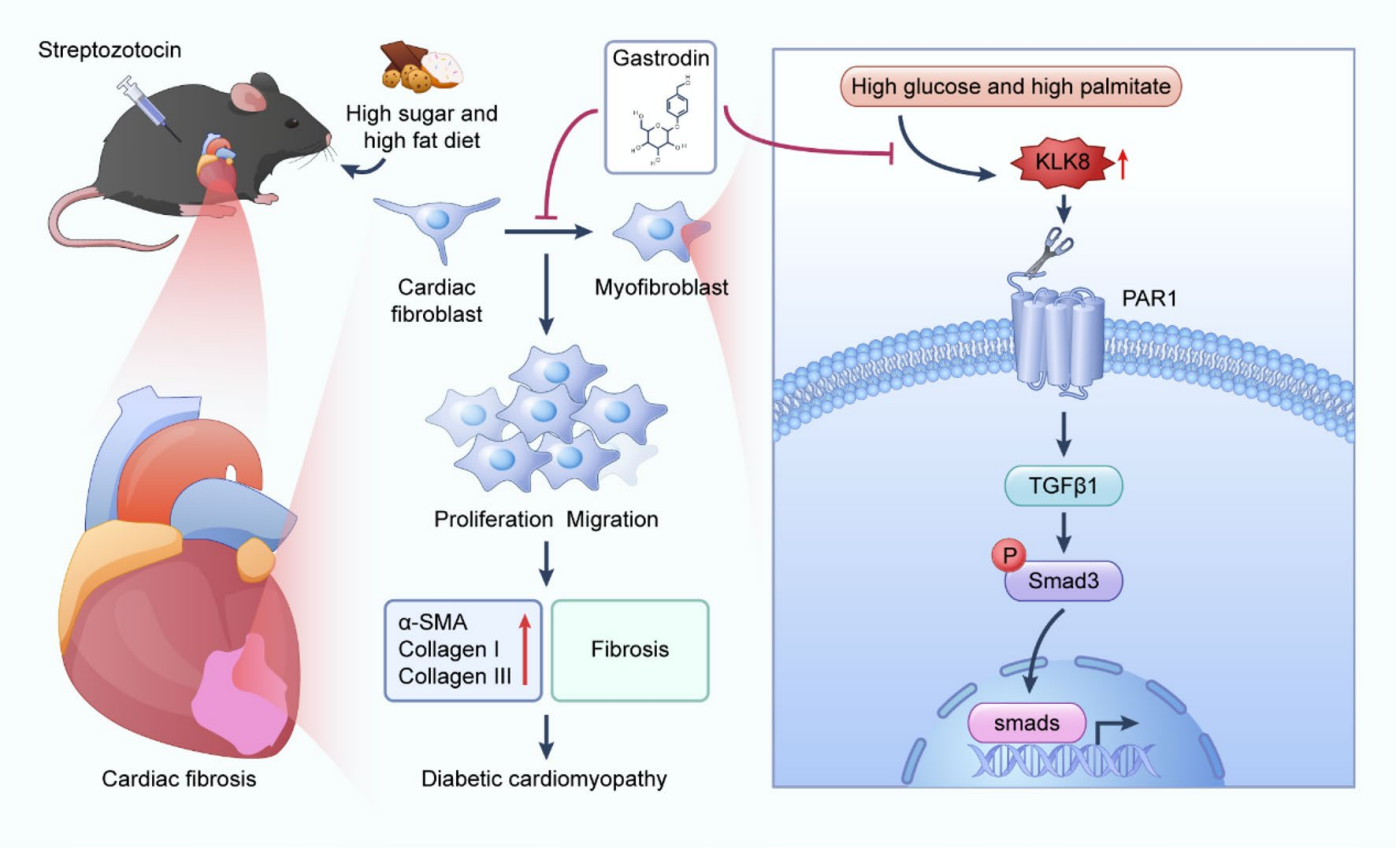

**Supplementary Information:**

The online version contains supplementary material available at 10.1186/s13020-024-01035-4.

## 1. Introduction

More than 90% of people with diabetes have type 2 diabetes (T2DM) with severe complications that usually lead to high mortality rates, characterized by relative pancreatic β-cell dysfunction, insulin resistance (IR) hyperglycemia, dyslipidemia, and uncontrolled protein and glucose metabolism; diabetic cardiomyopathy (DCM) is an extremely important complication of T2DM [1, 2]. DCM occurs due to the dysregulation of glucose and lipid metabolism associated with diabetes mellitus [3]. It is characterized by adverse structural remodeling factors, such as cardiac hypertrophy, myocardial fibrosis, early-onset diastolic dysfunction, and late-onset systolic dysfunction. When the heart suffers an injury, fibroblasts remodel the heart by increasing the amount of collagen and other components in the extracellular matrix, but if the degree of injury is severe, excess collagen can lead to fibrosis and dysfunction [4]. Despite extensive studies on the mechanisms underlying the development and progression of DCM, the specific molecular and metabolic changes associated with DCM remain unknown, primarily because the pathogenesis of DCM is multifactorial. Although researchers have shown great interest in DCM and the development of new therapeutic approaches, effective therapeutic strategies are lacking. Therefore, assessing and targeting new signaling pathways related to DCM evolution has become a research hotspot. Information on these pathways can contribute to the prevention and treatment of DCM.

Among all diabetic complications, DCM, characterized by myocardial fibrosis and cardiomyocyte loss, is the leading cause of mortality and morbidity in diabetic patients; progressive cardiac fibrosis occurs in both diabetic animal models and diabetic patients [5]. Cardiac fibrosis occurs when the heart is exposed to stress (e.g., ischemic injury and chronic hypertension). As fibrosis leads to cardiac insufficiency, it needs to be treated using drugs, medical devices, or tissue transplantation [6].

Myocardial fibrosis greatly contributes to heart failure and ventricular wall stiffness in patients with DCM. Early identification of drugs that can control myocardial fibrosis in DCM patients is important to prevent or delay the progression of DCM to heart failure [7].

Gastrodin (GAS) is the main component of gastrodia, which is mainly used in the clinical treatment of epilepsy and headache [8]. However, Yu Bai et al. showed that GAS has hypoglycemic activity, which can significantly reduce fasting blood glucose in streptozotocin-induced diabetic mice and improve insulin resistance to treat diabetes [9]. Ye et al. reported that GAS can alleviate diabetes-induced complications, ameliorate diabetic nephropathy, and attenuate diabetic encephalopathy, suggesting that GAS can effectively treat cardiovascular diseases [10]. GAS has gained much attention in the treatment of DCM, such as in the improvement of myocardial hypertrophy, hypertension, and myocardial ischemia-reperfusion injury. Moreover, GAS has negligible effects on the functional and histological characteristics of mouse organs [11]. However, the molecular mechanisms by which GAS inhibits DCM are unknown. Elucidating these mechanisms may reveal new molecular targets for DCM and potential targets for treating cardiac fibrosis.

Tissue kinin-releasing enzyme-related peptidase 8 (KLK8), a trypsin-like serine protease initially cloned as a neuropeptidase in the mouse brain, is highly expressed in the heart and other organs during development [12]. KLK8 can mediate DCM, and an increase in KLK8 levels promotes the development of diabetic cardiac fibrosis; KLK8 inhibition may be an effective strategy against DCM [13]. However, the mechanism of action is not known.

KLK8 is a secreted serine protease that mainly hydrolyses substrate proteins and strongly regulates cellular function and tissue reconstruction [14].

KLK8 regulates cellular signaling through the cleavage and activation of protease-activated receptors (PARs), which contribute to various cellular effects, including cardiac fibrosis and the development of cardiac hypertrophy [15, 16]. Mammalian PARs are a subgroup of GPCRs and include four members. Among them, the functions of PAR 1 and PAR 2 have been widely studied in various diseases, such as cardiac hypertrophy [14] and colorectal cancer [17]. PAR1 has been the subject of large-scale drug discovery programs. New insights into the mechanism underlying the functions of PAR1, the discovery of improved strategies for modifying PAR1 function, and the identification of PAR1 modulators have provided new opportunities for targeted PAR1 therapy.

In this study, we evaluated the effect of GAS on DCM induced by an HSF diet combined with STZ in male C57BL/6 mice, as well as, the protective effects and mechanisms of GAS on cardiac fibroblast transformation into myofibroblasts induced by high glucose and high palmitic acid (HG-PA) in vitro. We also assessed whether GAS attenuates myocardial fibrosis through the KLK8/PAR1 signaling axis. We aimed to determine the potential of GAS as a therapeutic agent for treating DCM.

## 2. Materials and methods

### 2.1 Reagents and antibodies

Gastrodin (GAS) (G2129091, purity: ≥98%) was purchased from Shanghai Aladdin Biochemical Technology Co., Ltd. PAR1 (141136–83 − 6, 1:1000), pSmad3 (YT4334, 1:1000), Smad3 (HY-P80325, 1:1000), SCH79797 (HY-14993), FSLLRY-NH2 (HY-P1260), and TFLLR-NH2 (HY-P0226A) were purchased from MedChemExpress. KLK8 (Ab232839, 1:1000) was purchased from Abcam. α-SMA (14395–1-AP1:2000), TGF-β1 (21898–1-AP, 1:2000), collagen I (14695–1-AP, 1:2000), collagen III (22734–1-AP, 1:1000), β-actin (20536–1-AP, 1:10000), and GAPDH (10494–1-AP, 1:10000) were purchased from Proteintech. Fluorescein isothiocyanate (FITC)-labeled goat anti-rabbit secondary antibodies (BS10950, 1:200) and anti-rabbit (SC-2030) IgG secondary antibodies (1:5000) were purchased from BioWorld.

### 2.2 Animals and treatment

Healthy male C57BL/6 mice (18–22 g, 6–8 weeks), specific pathogen-free (SPF) were purchased from SPF (Beijing, China) Biotechnology Co. Ltd. (Production License No. SCXK 2019–0010). All experiments were approved by the Animal Ethics Committee (No. 2000904) of Guizhou Medical University. The animal experiments followed experimental ethics and complied with Chinese animal welfare regulations.

We randomly divided 100 mice into a normal control group (*n* = 20, normal maintenance diet) and an HSF diet group (*n* = 80, HSF diet). The mice in the normal control group were fed normal chow, while those in the HSF group were fed HSF chow (MolDiets, China, NBM10160) [18]. The control light source mimicked the normal conditions of the mice. After eight weeks of feeding, the mice were anesthetized with isoflurane (1–2%), and glucose was injected intraperitoneally (0.01 mL of glucose at a concentration of 20% per gram of body weight) to measure the fasting blood glucose (FBG) levels and insulin sensitivity of the mice by collecting blood samples from the tail vein at 0 min, 15 min, 30 min, 60 min, 90 min, and 120 min.

After the mice developed insulin resistance, STZ (30 mg/kg/day) was injected intraperitoneally three times into the mice in the HSF group. The mice in the normal control group were injected with an equal volume of saline. After one week of adaptive feeding, the FBG level of the mice was measured; FBG levels greater than 11.1 mM indicated that the T2DM mouse model was successfully constructed. The mice in the HSF group were randomly divided into four subgroups, including DCM, DCM + Met (metformin, 100 mg/kg/day), DCM + GAS.L (low-dose GAS, 50 mg/kg/day), and DCM + GAS.H (high-dose GAS, 100 mg/kg/day). The dosing group were administered by gavage once every two days for 12 weeks. The normal control and DCM groups were administered saline treatment. Metformin, a drug commonly used for treating T2DM, was used in this study as a positive control drug.

### 2.3 Examination by echocardiography

The mice were anesthetized with isoflurane (1–2%). After carefully removing the hair from the left chest, the geometry of the heart was evaluated from the parasternal long-axis view using a small animal VINNO6LAB ultrasonic diagnostic apparatus (VINNO, Suzhou, China) attached to a mouse ultrasonic probe. The image of the left ventricular area was recorded via M-mode echocardiography. Echocardiography was performed to examine cardiac function and ventricular structure using the following parameters: left-ventricular end-diastolic and left-systolic internal diameter (LVEDD and LVESD, respectively), left-ventricular end-diastolic posterior wall thickness (LVPWd) and left-ventricular end-diastolic and left-systolic volume (LVEDV and LVESV, respectively). Left ventricular area fractional shortening (FS) and ejection fraction (EF) were also recorded.

### 2.4 Sirius red staining

The myocardial interstitial collagen was quantified by the Sirius Red staining technique. Fresh heart tissues were fixed with 4% paraformaldehyde overnight and embedded in paraffin. The paraffin was subsequently cut into 6–8 mm thick sections and stained with Sirius Red to evaluate the distributions and areas of collagen fibers in the heart tissue. Images were acquired using a Nano Zoomer-SQ (Hamamatsu Photonics, Hamamatsu, Japan).

### 2.5 Histological and IHC staining

First, the samples were fixed with 4% paraformaldehyde and embedded in paraffin. Then, serial left ventricle cross-Sect. (7 μm thick) were deparaffinized and stained with picric acid Sirius Red. The sections were also incubated with diluted primary antibodies at 4 °C overnight. Other steps for IHC staining were performed following the protocol provided with the two-step IHC kit (PV-9000, Zhongshan Golden Bridge Biotechnology).

### 2.6 Hematoxylin and eosin (H&E) staining

Heart tissue was fixed with 4% paraformaldehyde for more than 36 h, dehydrated, and embedded in paraffin. Wax blocks were prepared and cut into Sect. (3 μm thick) using a paraffin slicer. After the paraffin sections were deparaffinized, H&E staining, dehydration, and sealing were performed. Finally, images were captured and analyzed under a microscope (Nikon, Tokyo, Japan).

### 2.7 Masson staining

The hearts fixed with paraformaldehyde (4%) were sectioned transversely at the mid-ventricular level and embedded in paraffin. Next, tissue Sect. (5 μm thick) were obtained, deparaffinized, and stained sequentially with potassium dichromate, hematoxylin, lichen red acid fuchsin, phosphomolybdic acid, and aniline blue. The sections were sealed after differentiation. Finally, images were acquired using a microscope (Nikon, Tokyo, Japan).

### 2.8 Docking

The three-dimensional structure of GAS was obtained from the PubChem database. The KLK8 (PDB id: O60259) protein structure was downloaded from the UniProt PDB protein database (www.uniprot.org) and subsequently visualized using the PyMOL software. The structure of GAS was processed by ChemDraw 3D. The combined energies of GAS and KLK8 were analyzed using AutoDock 4.2.

### 2.9 Cellular thermal shift assay (CETSA)

First, 5 × 10^5^ cardiac fibroblasts (CFs) were inoculated in 6 cm dishes overnight and then treated with DMSO or GAS for 2 h. The cells were washed twice and resuspended in 500 µL of PBS. The lysates were divided into eight portions and loaded into PCR tubes (50 µL/tube), after which the samples were grouped and treated with temperatures (40, 43, 46, 49, 52, 55, 58, and 61 °C) for 5 minutes and then cooled on dry ice. The lysate was transferred to a new tube and centrifuged at 20,000 ×*g* for 20 min at 4 °C. Then, the lysate was cooled on dry ice. To the supernatant, 3x SDS Sampling Buffer was added, and the mixture was analyzed by immunoblotting.

### 2.10 Treatment and processing of primary cardiac fibroblasts

Healthy SPF Sprague-Dawley (SD) rat (180–220 g, 8–12 weeks) were provided by Changsha Tianqin Biotechnology Co., Ltd. (Production License No. SCXK (Xiang) 2019–0014). Rat pups (age:1–3 days) were provided by Guizhou Medical University. This experiment was approved by the Animal Ethics Committee of Guizhou Medical University (No. 2000132). The hearts of neonatal rats were rapidly removed in a sterile environment after these rats were anesthetized by isoflurane. Ventricular tissues were isolated from the atria, and primary neonatal rat CFs were extracted, isolated, purified, identified, and cultured by trypsin digestion. The cells were starved in a medium containing 0.5% fetal bovine serum for 12 h and then spread on coverslips pre-coated with HG-PA, OE-KLK8, KLK8 siRNA, the PAR1 antagonist SCH79797, FSLLRY-NH2, or the PAR1 agonist TFLLR-NH2 or culture plates with different concentrations of GAS.

### 2.11 Cell migration assays

Cardiac fibroblasts (CFs) were spread on six-well plates, and then, the monolayers were wounded with micropipette tips. Photographs were taken at 0 and 48 h post-wounding using a Leica DMi1 microscope and analyzed using the ImageJ software.

### 2.12 3-[4,5-Dimethylthiazol-2-yl]−2,5-diphenyl tetrazolium bromide (MTT) assay

Cell viability was evaluated by the MTT assay based on the reduction of MTT (Sigma-Aldrich) by functional mitochondria to formazan, as described in another study [19].

### 2.13 Immunofluorescence

First, the cells were fixed with 4% paraformaldehyde for 15 min, permeabilized with 0.2% Triton X-100 for 15 min, and blocked with 2% bovine serum albumin for 30 min. Then, the fixed cells were incubated overnight at 4 °C with a diluted primary antibody against α-SMA (14395-1-AP, 1:500). Next, the cells were incubated with the appropriate secondary antibody for 1 h at 37 °C in the dark. Finally, the nuclei were stained with DAPI, and images were acquired using an inverted fluorescence microscope (Leica, Germany).

### 2.14 Transfection of the KLK8 plasmid

The plasmid pEX-3 containing the KLK8 gene (NM_26550) was constructed by GenePharma Corporation (Shanghai, China), and transfection was performed using the Lipofectamine™ 2000 Transfection Reagent (Thermo Fisher, NM_11668500) following the manufacturer’s protocol.

### 2.15 Transfection of small interfering RNA (siRNA)

The siRNA for KLK8 was designed and synthesized by GenePharma Corporation (Shanghai, China). The target sequences of the rat KLK8 siRNAs were 5′-CAACUGUGCAGAAGUGAAATT-3′ and 5′-UUUCACUUCUGCACAGUUGTT-3′, respectively. The scrambled sequences of the negative control siRNAs did not have any specific targets. The cells were then transfected using the Lipofectamine™ 2000 in vitro transfection reagent (Thermo Fisher, NM_11668500) following the manufacturer’s protocol.

### 2.16 Quantitative real-time PCR

Total RNA from the myocardium or CFs was extracted using TRIzol reagent (Takara) and reverse-transcribed to cDNA using reverse transcriptase (Invitrogen). Real-time quantitative PCR was performed using a real-time PCR detection system (Bio-Rad Laboratories). The SYBR Green dye (Roche Ltd., Basel, Switzerland) was used to detect the PCR products. The annealing temperature was set at 55–65 °C, and 45 amplification cycles were performed. The melting temperature of the PCR products was set at 60–95 °C. Relative quantification of gene expression was performed using the arithmetic formula Comparative Ct (Threshold Cycling) (2^−ΔΔCt^). The primer sequences used in this study are shown in Table 1.

**Table 1:** The list of primer sequences used in the study

Gene	Species	Forward prime	Reverse primer
KLK8	Mus	TCCTGGAAGGTCGAGAGTGTA	CTCCGGCTGATCTCTGCTC
KLK8	RAT	TCCAGGTGGCTCGGTCTA	CGATGGGCTTCACTTTGTC
α-SMA	Mus	GTCCCAGACATCAGGGAGTAA	TCGGATACTTCAGCGTCAGGA
α-SMA	RAT	GGCTATTCCTTCGTGACTACTG	AGCAGTGGCCATCTCATTT
TGF-β1	Mus	CCACCTGCAAGACCATCGAC	CTGGCGAGCCTTAGTTTGGAC
TGF-β1	RAT	GCAACAATTCCTGGCGTTA	TTCCGTCTCCTTGGTTCAG
COI	Mus	ACAGTCGCTTCACCTACAGC	GGGTGGAGGGAGTTTACACG
COI	RAT	ACTGGTACATCAGCCCAAAC	GGAACCTTCGCTTCCATACTC
COIII	Mus	ACGTAAGCACTGGTGGACAG	CAGGAGGGCCATAGCTGAAC
COIII	RAT	GAAGTCTCTGAAGCTGATGGG	GGCCTTGCGTGTTTGATATTC

### 2.17 Western blotting

Heart tissue or primary neonatal rat cardiomyocytes were lysed using RIPA lysis buffer (Beijing Sola Biotechnology Co., Ltd.) supplemented with 1× protease inhibitor cocktail solution and PMSF (Beijing Sola Biotechnology Co., Ltd.). The total protein concentration was determined using a BCA protein assay kit (Beijing Sola Biotechnology Co., Ltd.). After the samples were blocked with 5% skim milk in Tris-buffered saline containing 0.1% Tween 20, SDS-PAGE was performed, after which the proteins were transferred onto a polyvinylidene difluoride (PVDF) membrane. Then, the membrane was blocked with 5% skim milk in Tris-buffered saline containing 0.1% Tween 20. The membrane was subsequently incubated overnight with the primary antibodies at 4 °C. The protein bands were visualized using an enhanced chemiluminescence (ECL) solution (New Cell Life Science Co., Ltd., China) and scanned with a Bio-Rad ChemiDoc XRS + imaging system (USA). The data were statistically analyzed using the ImageJ software.

### 2.18 Statistical analysis

The data were expressed as the mean ± SEM. The differences between experimental groups were evaluated by one-way ANOVA with a two-sided t-test using GraphPad Prism 10.1.2. All differences were considered to be statistically significant at *P* < 0.05.

## 3. Results

### ***GAS rescues impaired cardiac function and attenuates cardiac fibrosis in DCM mice***,*** with possible involvement of KLK8***

**Fig. 1:**
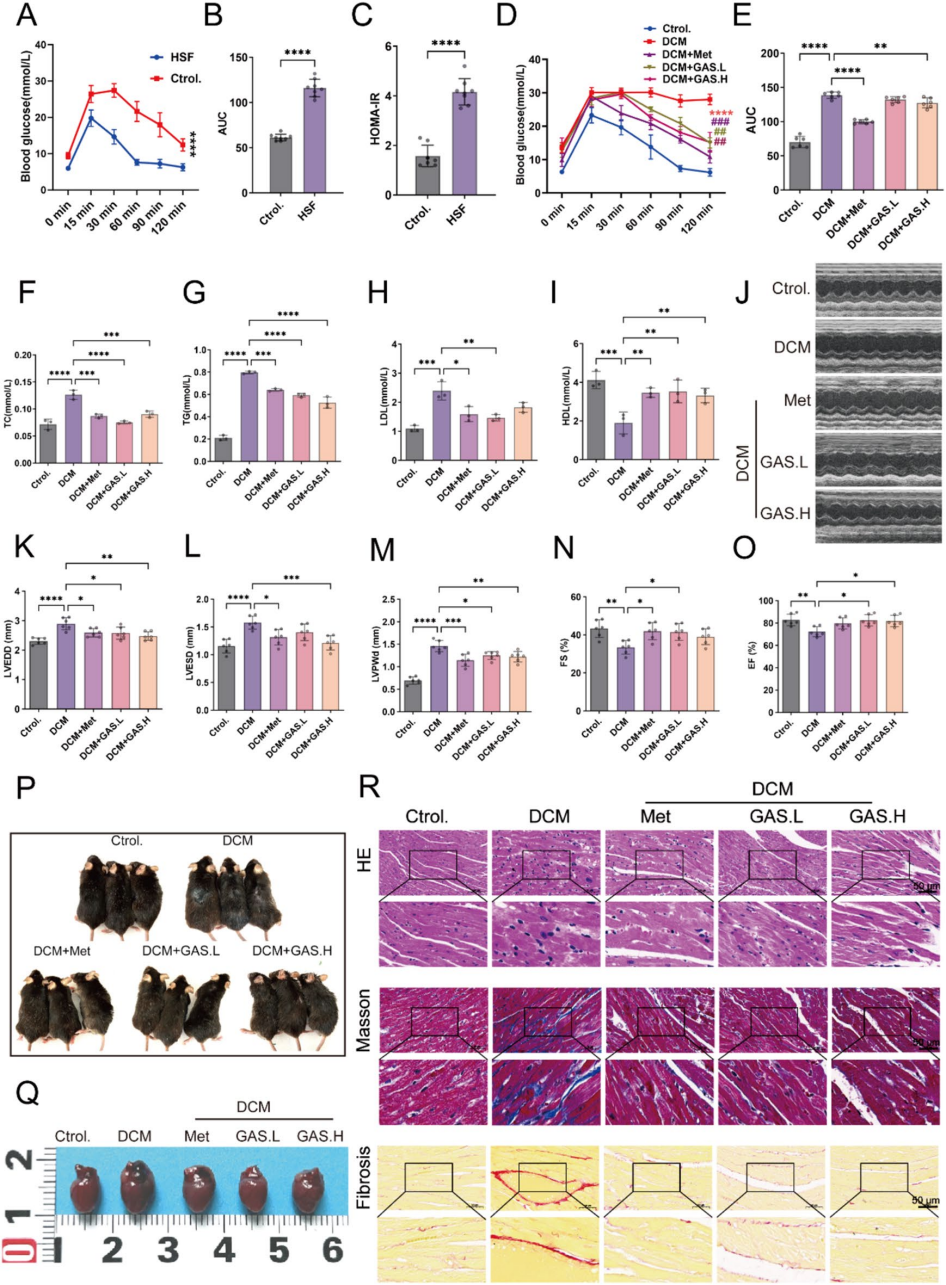
Impaired cardiac function and myocardial pathological changes in DCM mice were ameliorated by GAS.  After one week of acclimation, the mice were fed an HSF diet for eight weeks, whereas the control group received a normal diet.  **A** Fasting blood glucose levels in the mice. **B** Area under the curve of blood glucose decline. **C** Insulin sensitivity in mice. After insulin resistance occurred, the HFS group was administered intraperitoneal injections of STZ (30 mg/kg/day) three times a day for two days. The normal control group was injected with an equal volume of saline. After DCM was established in the mice, **D–R** was administered by gavage once every two days for 12 weeks. **D** Fasting blood glucose levels in DCM mice; * P  < 0.05 vs. control; # P  < 0.05 vs. DCM. **E** Area under the curve of blood glucose decline. **F–I**. Lipid quadruple content TC, TG, LDL, and HDL assays. **J–O** Small-animal ultrasonography was performed to detect the parameters of left ventricular end-diastolic and end-systolic internal diameters (LVEDD and LVESD), left ventricular posterior wall thickness at end-diastole (LVPWd), fractional shortening (FS), and ejection fraction (EF) (n  = 6). **P–Q** Degloving phenomenon and heart size in mice. **R** H&E staining, Masson staining, and Sirius Red staining (n  = 3). The data are expressed as the mean ± SEM of three or more independent experiments; * p  < 0.05, ** p  < 0.01, *** p  < 0.001, and **** p  < 0.0001.

To assess the role of GAS in the development of diabetes-associated cardiomyopathy, we constructed a DCM mouse model via an HSF diet combined with low-dose STZ induction. Fasting blood glucose (FBG) levels were measured in blood samples from the tail vein after administering the HSF diet for eight weeks. Compared to that in the normal chow-fed control mice, the blood glucose level in the mice of the HSF group was significantly elevated to produce glucose tolerance, and they showed insulin resistance (Fig. 1A–C). Next, we administered STZ intraperitoneally to the mice in the HSF group and saline to those in the normal group. After 12 weeks of gavage, to assess the physiological effects of GAS treatment on systemic glucose-lipid metabolic homeostasis, we examined the changes in fasting blood glucose and lipid levels. Similar to metformin treatment, GAS treatment had a hypoglycemic effect on DCM mice (Fig. 1D, E). The serum levels of total cholesterol (TC), triglycerides (TG), and low-density lipoprotein cholesterol (LDL) were significantly elevated, and high-density lipoprotein cholesterol (HDL) was significantly decreased in DCM mice; GAS treatment significantly decreased TC, TG, and LDL levels and increased HDL levels (Fig. 1F–I). These findings suggested that GAS treatment can restore glucose homeostasis and decrease lipogenesis in mice.

In the next step, we performed echocardiography analysis to evaluate cardiac function. Compared to the mice in the normal group, those in the DCM group presented greater left ventricular end-diastolic and end-systolic sizes, as well as, a decline in contractile function. Mice treated with GAS exhibited significantly reduced left ventricular end-diastolic and end-systolic sizes, along with higher FS and EF values (Fig. 1J–O). These findings indicated that GAS can effectively ameliorate impaired cardiac function in DCM mice. Moreover, the mice in the GAS-treated group presented reduced hair loss and cardiac hypertrophy (Fig. 1P–Q).

To assess the contribution of GAS to the development of myocardial fibrosis in DCM, histological examination of mouse heart tissues was performed, and the images revealed that the myocardial cells in the control group were densely arranged without any discernible cell death. In contrast, the DCM group exhibited disordered myocardial cell arrangement, cellular hypertrophy, and significant infiltration of inflammatory cells. Masson’s trichrome staining and Azure Red staining revealed significant blue collagen deposition and high levels of fibrosis in the cardiac interstitium of the mice in the DCM group; GAS treatment significantly ameliorated these pathological changes (Fig. 1R).

**Fig. 2:**
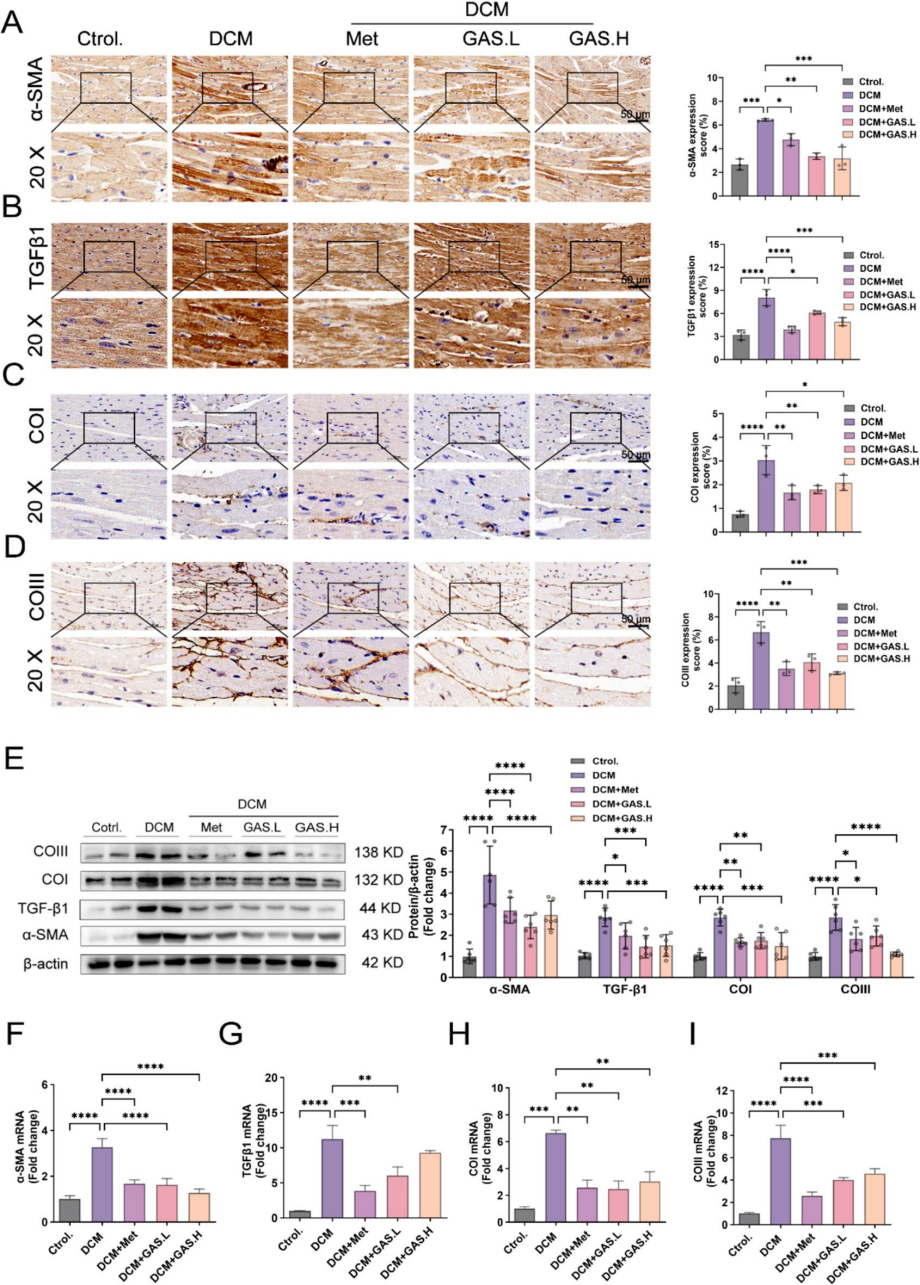
GAS ameliorates myocardial fibrosis in DCM mice. **A–D** Immunohistochemical staining of the myocardial fibrosis proteins α-SMA, TGFβ1, Collagen I, and Collagen III; magnification (20×) (n  = 6). **E** Immunoblotting for the myocardial fibrosis proteins α-SMA, TGFβ1, Collagen I, and Collagen III (n  = 6). **F–I** Myocardial fibrosis gene α-SMA, TGFβ1, Collagen I, and Collagen III mRNA expression levels (n  = 3). The data are expressed as the mean ± SEM of three or more independent experiments; * p  < 0.05. ** p  < 0.01, *** p  < 0.001, and **** p  < 0.0001.

We next determined the expression of fibrosis-associated proteins and mRNAs in the myocardium by immunohistochemical staining, protein blotting, and real-time fluorescence quantitative PCR. The results of the immunohistochemical staining and immunoblotting experiments showed that the level of expression of fibrosis-associated proteins in the DCM mice was significantly greater than that in the control mice (Fig. 2A–E), and GAS treatment decreased the level of expression of fibrosis-associated proteins in the hearts of DCM model mice. The real-time fluorescence quantitative PCR results confirmed these findings (Fig. 2F–I).

**Fig. 3:**
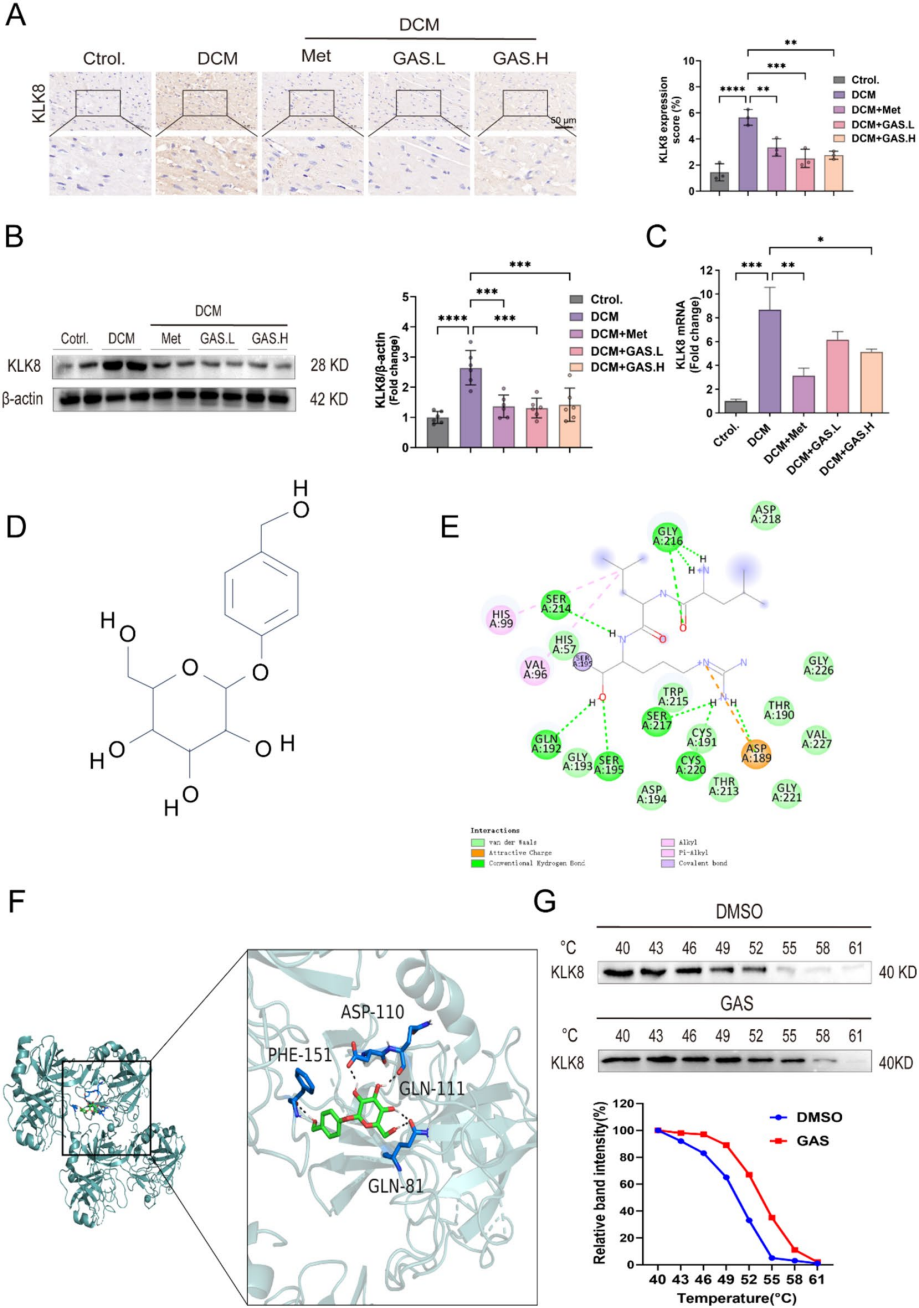
GAS treatment attenuated the increased expression of KLK8 in the diabetic myocardium.  **A** Immunohistochemical staining and quantification of elevated levels of KLK8 in the myocardium. **B** Immunoblot of KLK8 in the heart. **C** The level of expression of KLK8 mRNA in the heart. **D** Chemical structural formula of GAS. E-F. Molecular docking data of GAS with the KLK8 protein was analyzed using the automated docking software. **E** Two-dimensional interaction. **F** Three-dimensional interaction. **G** Cellular thermal transfer assay (CETSA) and immunoblotting analysis were performed with cells treated with 5 µM GAS and DMSO at different temperatures. The data are expressed as the mean ± SEM of three or more independent experiments; *p  < 0.05, **p  < 0.01, ***p  < 0.001, and ****p  < 0.0001.

Some studies have reported that the overall absence of KLK8 can significantly alleviate endothelial-mesenchymal transition and cardiac fibrosis in individuals with diabetes, while transgenic overexpression of KLK8 can lead to myocardial mesenchymal/perivascular fibrosis and endothelial-mesenchymal transition [13]. The expression of KLK8 increased in the left ventricle of cardiac hypertrophy model mice. Intracardiac adenovirus-mediated and transgenic-mediated increase in KLK8 led to cardiac hypertrophy in vivo [14]. These findings suggested that the upregulation of KLK8 plays a role in the progression of diabetes-induced endothelial mesenchymal transition and cardiac fibrosis. In this study, KLK8 expression in cardiac tissues revealed a substantial increase in KLK8 staining in the myocardium of mice in the DCM group compared to that in the mice from the control group (Fig. 3A). Additionally, immunoblotting and real-time fluorescence quantitative PCR assays confirmed an increase in the levels of KLK8 proteins and mRNAs in the myocardia of diabetic mice (Fig. 3B-C); however, their levels decreased substantially after GAS was administered. To summarize, GAS rescued DCM-induced cardiac dysfunction and mitigated the onset of cardiac fibrosis in DCM mice, potentially through KLK8.

We used the automated docking software to analyze the molecular docking data of GAS (Fig. 3D) on the KLK8 protein. GAS effectively bound to the active pocket of the KLK8 protein, engaging in strong spontaneous hydrogen bonding interactions and hydrophobic interactions with the protein binding pocket. After the structure of GAS was processed using ChemDraw 3D, the compound was found to bind primarily to the active site of the protein through hydrogen bonding. The compound engaged in hydrogen bonding interactions with the amino acids PHE-151, ASP-110, GLN-111, and GLN-81 of the protein (Fig. 3E-F), which indicated that GAS can interact with the KLK8 protein. To confirm this result, we performed a cellular thermal shift assay (CETSA), which is conducted to study the binding between drugs and targets in a cellular environment. The results revealed that compared to DMSO, GAS significantly increased the thermal stability of KLK8 even at relatively high temperatures (Fig. 3G), indicating that GAS binds to KLK8. To summarize, these results indicated that GAS has a wide range of pharmacological activities and plays a broad role in treating DCM.

### 3.2 GAS attenuates cell differentiation, collagen synthesis, and cell migration in HG-PA-exposed CFs

**Fig. 4:**
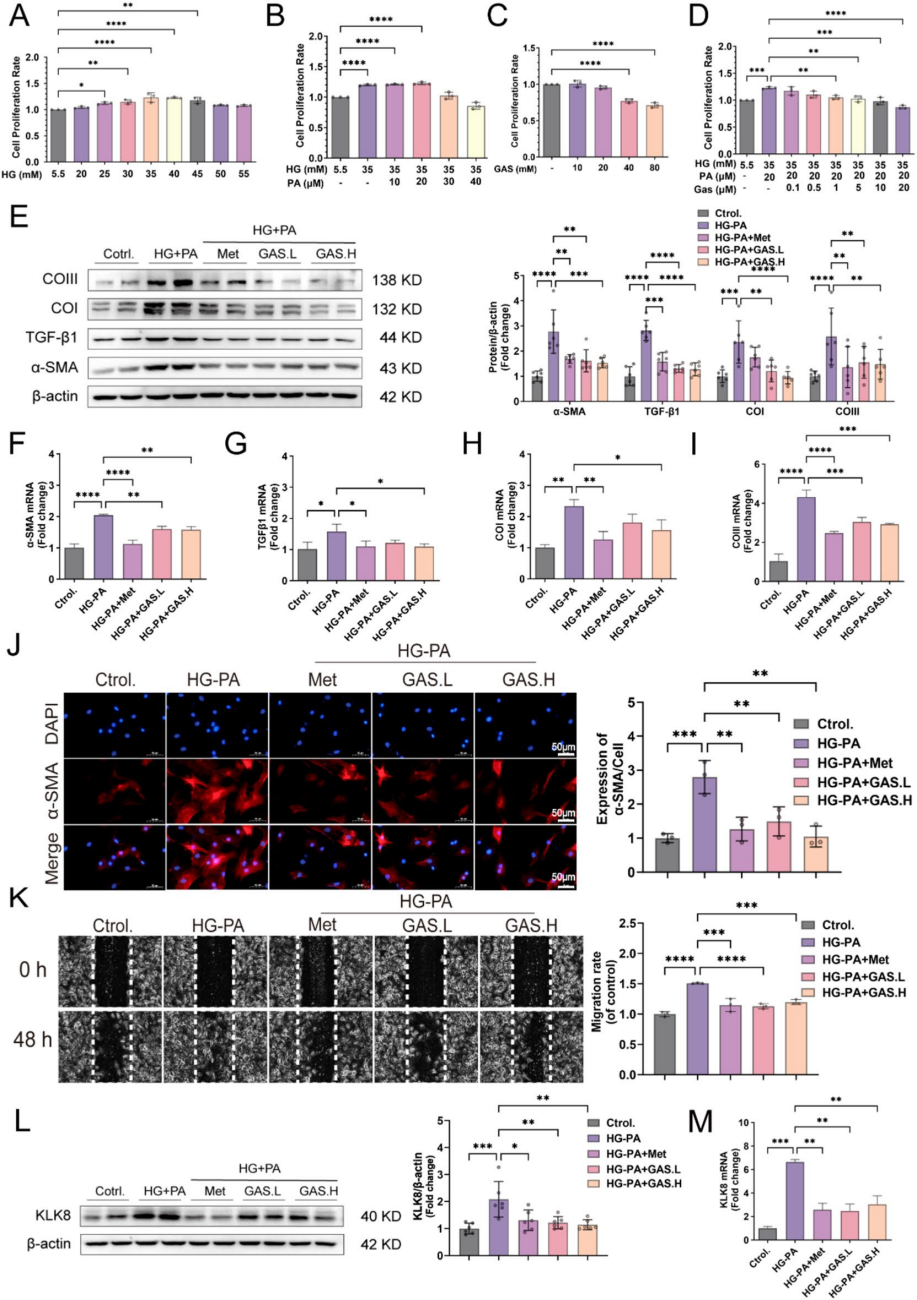
GAS inhibits the differentiation, collagen synthesis, and migration of HG-PA-exposed CFs.  HG-PA (35 mM or 20 µM) with or without GAS (1 µM or 5 µM) treatment for 48 h. **A** Value-added effects of different concentrations of HG exposure in CFs. **B** Value-added effects of HG combined with various PA doses on CFs. **C** Safe concentrations of CFs for GAS exposure. **D** Rescue effects of GAS in HG-PA. **E** Immunoblotting of the fibrosis-associated proteins α-SMA, TGFβ1, Collagen I, and Collagen III. **F–G** Real-time fluorescence quantitative PCR was performed to detect the mRNA expression levels of α-SMA, TGFβ1, Collagen I, and Collagen III. **J** Representative images and quantitative results of the immunofluorescent analysis of α-SMA, α-SMA (red), and DAPI (blue) staining of the cell nuclei; scale bar = 50 μm. K. Wound healing assay at 0 and 48 h. Images were captured to assess cell migration ability by measuring the distance between advancing edges of the cells; scale bar = 50 μm. L. Immunoblotting of KLK8 proteins. M. Real-time fluorescence quantitative PCR was performed to detect KLK8 mRNA expression levels. The data are expressed as the mean ± SEM of three independent experiments; *p  < 0.05, **p  < 0.01, ***p  < 0.001, and ****p  < 0.0001.

Palmitic acid (PA) is the most abundant saturated free fatty acid (FFA) and is involved in cardiac fibrosis in T2DM [20]. The main feature of myocardial fibrosis is the activation of CFs, which causes them to proliferate, migrate, and synthesize collagen [21]. Therefore, we used HG-PA-exposed CFs in vitro to mimic DCM caused by an imbalance in glycolipid metabolism in vivo. The most effective value-added conditions were first identified by the HG value-added concentration combined with various PA doses, and a follow-up assessment was performed using the HG (35 mM) + PA (20 µM) concentration for 48 h (Fig. 4A, B). The safe concentration of GAS in CFs was then tested, it was introduced into HG-PA-exposed CFs at 1 µM or 5 µM (Fig. 4C–D) for subsequent rescue testing. The effects of GAS on HG-PA-enhanced cell differentiation, collagen synthesis, and cell migration abilities were tested. As the fibrogenic growth factor in cardiac fibrosis, TGF-β1, exposes fibroblasts to myofibroblasts, leading to high expression of the α-SMA protein [22], our analysis involved assessing α-SMA and TGFβ1 at the protein and mRNA levels through protein blotting and real-time fluorescence quantitative PCR assays to determine the differentiation potential of CFs. Following HG-PA treatment for 48 h, a substantial increase in α-SMA protein and mRNA levels was recorded, and this increase was suppressed after GAS was administered (Fig. 4E–I). These findings suggested that GAS effectively hindered the transdifferentiation of HG-PA-exposed CFs. The inhibitory effect of GAS was confirmed by α-SMA immunofluorescence analysis (Fig. 4J). The extracellular matrix protein collagen is a major contributor to the shift of fibroblasts to myofibroblasts. We investigated collagen synthesis, a key process involved in the conversion of fibroblasts to myofibroblasts. Quantitative assessment of the Collagen I and Collagen III proteins and mRNAs showed an increase in their level of expression following HG-PA exposure; these changes were reversed upon preincubation with GAS (Fig. 4E, H, and I). The optimal activity and abundance of myofibroblasts play crucial roles in impeding the progression to severe fibrosis and facilitating recovery from such states [23]. When the migratory potential of CFs was evaluated through a wound healing assay, preincubation with GAS significantly impeded the migration of HG-PA-exposed CFs (Fig. 4L). These findings highlighted the direct increase in cell differentiation, collagen synthesis, and cell migration in HG-PA-exposed CFs due to the inhibitory effects of GAS. The pivotal role of KLK8 in diabetes-associated cardiomyopathy was also highlighted through our results [13]. In vivo models involving mice and in vitro experiments involving HG-PA-exposed CFs revealed a substantial increase in KLK8 protein and mRNA levels, an effect that was mitigated by GAS (Figs. 3B–D and 4L–M). These results suggested that KLK8 can mediate the influence of GAS on myocardial fibrosis.

### 3.3 KLK8 mediates differentiation, collagen synthesis, and cell migration in HG-PA-exposed CFs

**Fig. 5:**
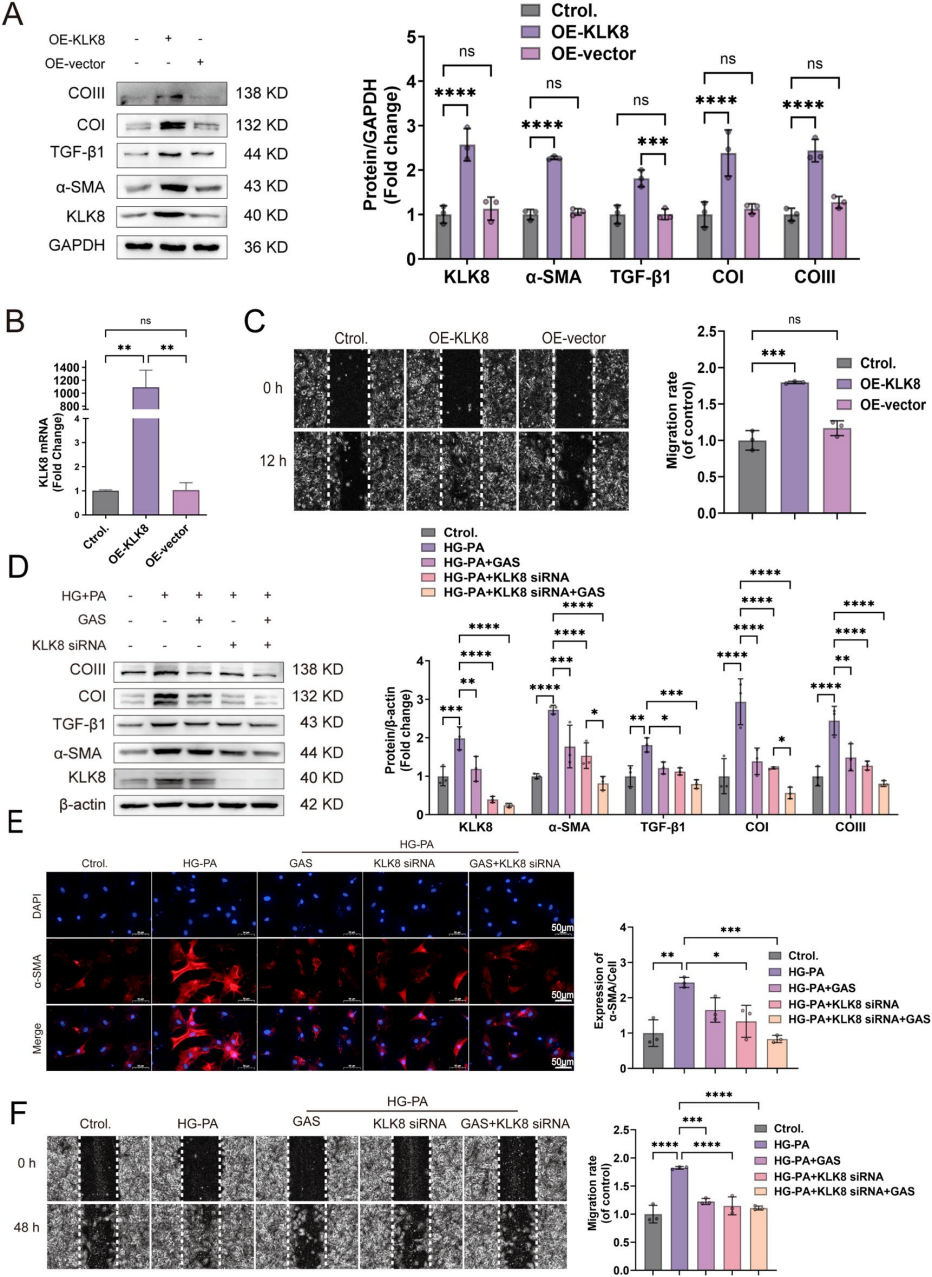
KLK8 mediates the differentiation, collagen synthesis, and migration of HG-PA-exposed CFs.  **A–C** OE-KLK8-transfected CFs for 12 h. **A** OE-KLK8 increased the expression of the fibrosis-related proteins KLK8, α-SMA, TGFβ1, Collagen I, and Collagen III in CFs. **B** KLK8 mRNA was highly expressed in OE-KLK8-exposed CFs. **C** OE-KLK8-exposed CFs. The cell migration capacity was measured via a wound healing assay; scale bar = 50 μm. **D–F** CFs were transfected with KLK8 siRNA for 8 h, exposed to HG-PA, and then incubated for 40 h with or without GAS (5 µM). **D** Immunoblots showing the expression of the fibrosis-associated proteins KLK8, α-SMA, TGFβ1, Collagen I, and Collagen III. **E** Immunofluorescence staining of α-SMA is shown; α-SMA (green) and cell nuclei were restained with DAPI (blue); scale bar = 50 μm. **F** Wound healing assay; scale bar = 50 μm. The data are expressed as the mean ± SEM of three independent experiments; *p  < 0.05, **p  < 0.01, ***p  < 0.001, and ****p  < 0.0001.

To determine whether KLK8 mediates the function of CFs, we introduced the OE-KLK8 plasmid. First, we examined whether overexpressing KLK8 increases CF differentiation, collagen synthesis, and cell migration. We found that in the CFs transfected with the OE-KLK8 plasmid, the levels of KLK8 mRNA and protein increased (Fig. 5A, B). Fibrosis-related protein levels and CF migration ability increased (Fig. 5C), indicating that KLK8 alone can induce CF differentiation, collagen synthesis, and cell migration.

Next, we investigated whether the upregulation of KLK8 mediates HG-PA exposure-induced differentiation, collagen synthesis, and cell migration in CFs. Exposure to KLK8 siRNA not only significantly decreased the expression of KLK8 in CFs but also prevented the upregulation of KLK8 induced by exposure to HG-PA. The increase in fibrosis-related protein levels, greater α-SMA immunofluorescence intensity, and higher migration ability in CFs following exposure to HG-PA were reversed by KLK8 siRNA, and this effect was further enhanced when KLK8 siRNA was used simultaneously with GAS (Fig. 5D–F). These findings indicated that KLK8 mediates CF differentiation, collagen synthesis, and cell migration induced by HG-PA exposure.

### 3.4 GAS reduces the differentiation, collagen synthesis, and migration of HG-PA-exposed CFs by inhibiting KLK8 upregulation

**Fig. 6:**
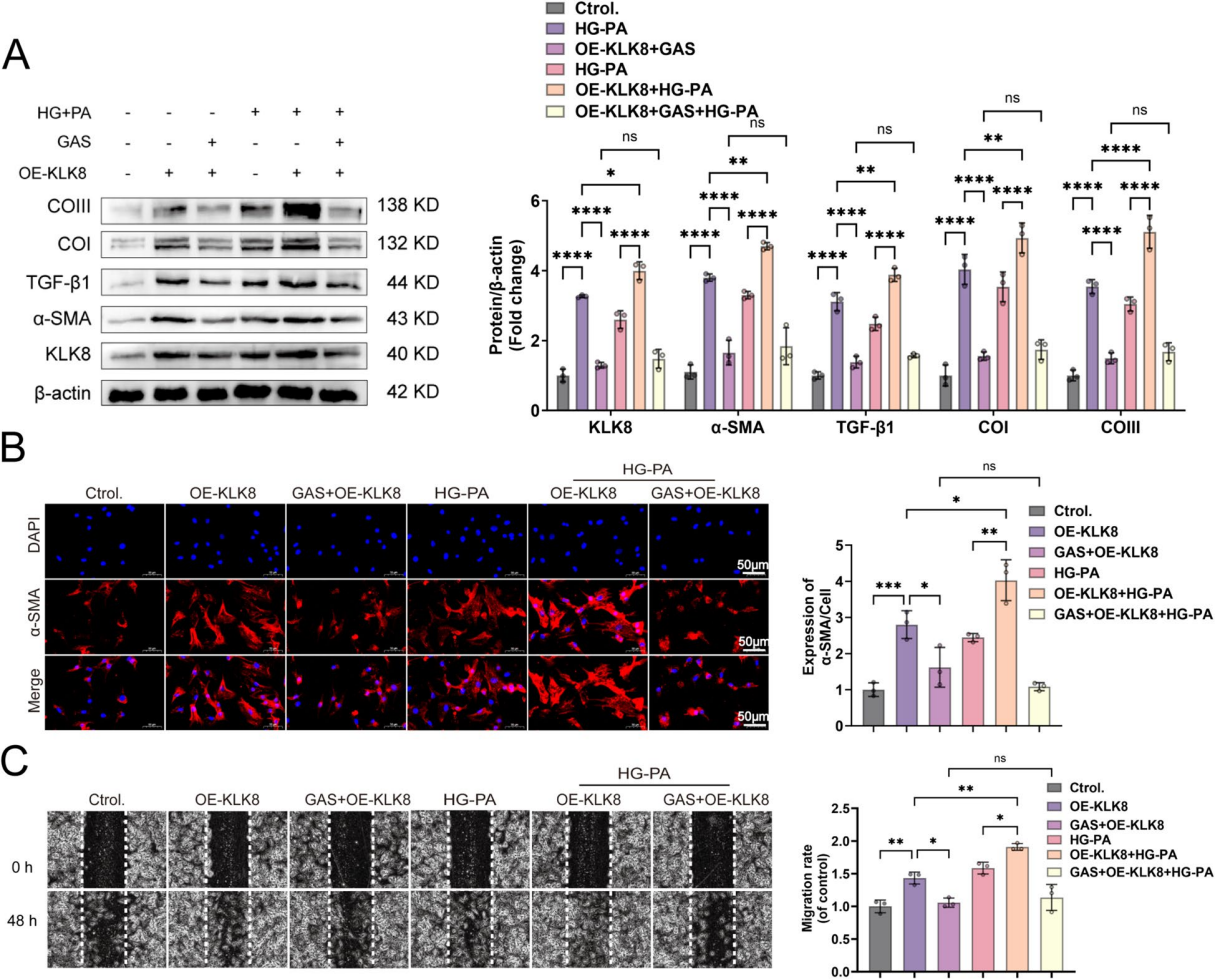
GAS reduces the differentiation, collagen synthesis, and migration of HG-PA-exposed CFs by inhibiting KLK8 expression. CFs were transfected with OE-KLK8 with or without GAS (5 µM) for 12 h and then exposed to HG-PA with or without HG-PA for 36 h. **A** Expression of the fibrosis-associated proteins KLK8, α-SMA, TGFβ1, Collagen I, and Collagen III. **B** Immunofluorescence staining revealed that α-SMA (green) was reduced, and the nuclei of the cells were stained with DAPI (blue), scale bar = 50 μm. **C** Wound healing assay; scale bar = 50 μm. The data were expressed as the mean ± SEM of three independent experiments; *p < 0.05, **p < 0.01, ***p < 0.001, and ****p < 0.0001.

To determine whether the reduction in KLK8 contributes to the protective effects of GAS on HG-PA-exposed CFs, we evaluated their differentiation, collagen synthesis, and cell migration. CFs were pretreated with or without GAS for 12 h, followed by exposure to HG-PA using OE-KLK8. Compared to the control, GAS significantly inhibited the upregulation of fibrotic proteins in OE-KLK8-exposed CFs. HG-PA-induced CF cell differentiation, collagen synthesis, and cell migration were blocked when GAS was coincubated with OE-KLK8 (Fig. 6A-C). These findings suggested that GAS attenuates CF differentiation, collagen synthesis, and cell migration by inhibiting KLK8 upregulation to reduce exposure to HG-PA.

### ***KLK8 activates PAR1 receptors***,*** which are involved in differentiation***,*** collagen synthesis***,*** and cell migration in HG-PA-exposed CFs***

**Fig. 7:**
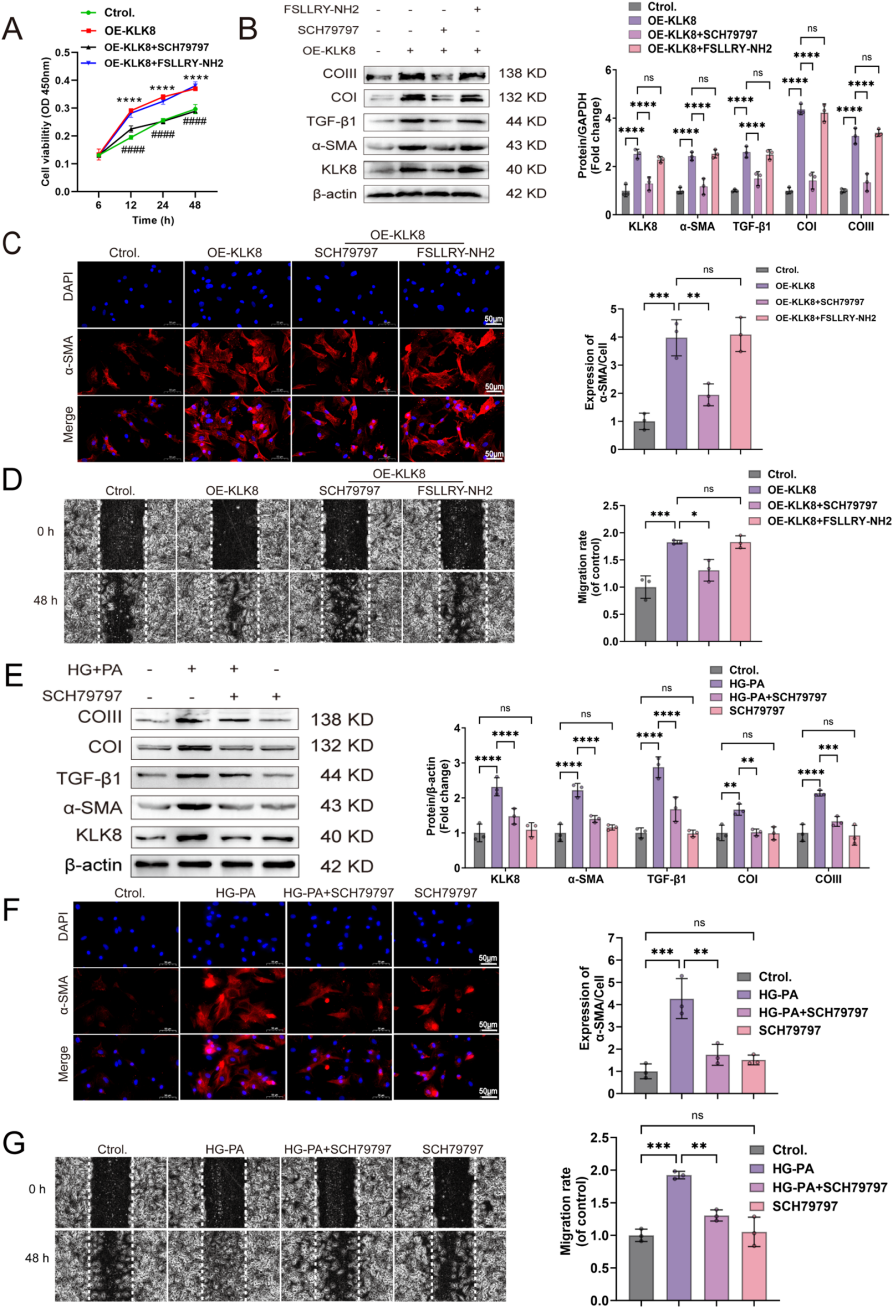
KLK8 exerts biological effects through the activation of the PAR1 receptor, which is involved in HG-PA-induced differentiation, collagen synthesis, and the migration of CFs.  **A–D** OE-KLK8-treated CFs were continuously transfected for 12 h. Interventions were performed using the PAR1 and PAR2 antagonists SCH79797 and FSLLRY-NH2. E-G. The PAR1 antagonist SCH79797 was used to treat HG-PA-exposed CFs. **A** OE-KLK8-overexpressing CFs were treated with the PAR1 antagonist SCH79797 (10 µM) or the PAR2 antagonist FSLLRY-NH2 (1 µM). The MTT assay was performed to determine the proliferation rate of the cells; *P  < 0.05 vs. control; # P  < 0.05 vs. OE-KLK8. **B** Immunoblotting of KLK8 and the fibrosis-associated proteins α-SMA, TGFβ1, Collagen I, and Collagen III. **C** Immunofluorescence staining of α-SMA (green); the nuclei were restained with DAPI (blue); scale bar = 50 μm. **D** Wound healing assay; scale bar = 50 μm. **E** Immunoblotting of KLK8 and the fibrosis-associated proteins α-SMA, TGFβ1, Collagen I, and Collagen III. **F** Immunofluorescence staining of α-SMA (green). The cell nuclei were restained with DAPI (blue); scale bar = 50 μm. **G** Wound healing assay; scale bar = 50 μm. The data are expressed as the mean ± SEM of three independent experiments; *p  < 0.05, **p  < 0.01, ***p  < 0.001, and ****p  < 0.0001.

KLKs regulate cellular functions by cleaving and activating members of the protease-activated receptor (PAR) family [24, 25]. Among them, PAR1 and PAR2 play pivotal roles and have been implicated in various diabetic complications [26, 27]. To elucidate the mechanism of action of KLK8 in cardiac fibroblasts (CFs), we proposed that KLK8 exerts its effects by modulating PAR1 and PAR2 receptors in CFs, leading to CF cell differentiation, collagen synthesis, and cell migration. Therefore, we pretreated the cells with OE-KLK8 for 12 h, and intervention with the antagonists PAR1, PAR2, SCH79797, and FSLLRY-NH2 showed that CF proliferation was affected by OE-KLK8 (Fig. 7A), along with an increase in fibrosis-related proteins; these changes were reversed by the PAR1 antagonist SCH79797 but not the PAR2 antagonist FSLLRY-NH2 (Fig. 7B). Immunofluorescence staining of α-SMA and wound healing experiments confirmed this effect (Fig. 7C-D). Our findings revealed that KLK8 exerts its biological effects by activating the PAR1 receptor in CFs. Next, we investigated whether PAR1 is involved in the differentiation, collagen synthesis, and migration of CFs exposed to HG-PA. The increase in KLK8 in CFs exposed to HG-PA was inhibited by the PAR1 antagonist SCH79797 (Fig. 7), whereas treatment with the PAR1 antagonist SCH79797 alone did not affect the cells (Fig. 7E). Additionally, the increase in the expression of fibrosis-related proteins in CFs exposed to HG-PA was suppressed by the PAR1 antagonist SCH79797 (Fig. 7E). Immunofluorescence staining of α-SMA and wound healing experiments confirmed these results (Fig. 7F–G), suggesting that the cell differentiation, collagen synthesis, and migration of CFs exposed to HG-PA require PAR1.

### GAS attenuates fibrosis in CFs by inhibiting the KLK8-PAR1 signaling axis, a process in which TGF-β1/Smad3 is involved

**Fig. 8:**
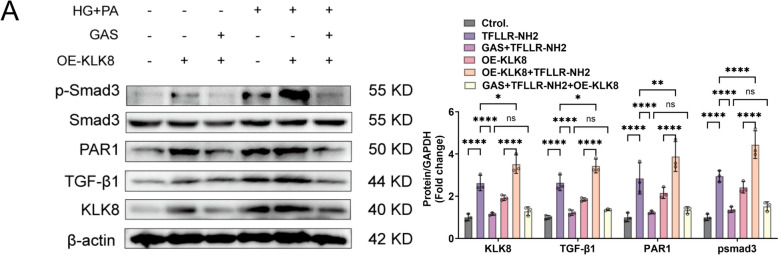
GAS attenuates fibrosis in CFs by inhibiting the KLK8-PAR1 signaling axis, a process in which TGF-β1/smad3 is involved.  CFs were transfected with OE-KLK8 with or without GAS (5 µM) treatment for 12 h, followed by 36 h of exposure in the presence or absence of HG-PA. **A** Immunoblotting of KLK8, TGFBβ1, PAR1, and Smad3 proteins. The data are expressed as the mean ± SEM of three independent experiments; *p  < 0.05, **p  < 0.01, ***p  < 0.001, and ****p  < 0.0001.

We next assessed whether GAS attenuated the increase in PAR1 expression by preventing the upregulation of KLK8, thus inhibiting the fibrotic effect of HG-PA-induced CFs. We first pretreated CFs with OE-KLK8 in the presence or absence of GAS for 12 h and then exposed them to HG-PA. GAS inhibited the expression of the PAR1 protein in OE-KLK8-exposed CFs compared to its expression in the normal group and attenuated the expression of the PAR1 protein in HG-PA-exposed CFs when GAS was preincubated with OE-KLK8. The typical TGF-β1 signaling pathway via Smad3 phosphorylation was enhanced in OE-KLK8-exposed CFs, and this effect was further enhanced when OE-KLK8 was exposed concomitantly to HG-PA; these changes were significantly inhibited by preincubation of GAS with OE-KLK8 (Fig. 8B). Our results suggested that GAS inhibition suppresses TGF-β and downstream Smad3 signaling and this effect is probably achieved by inhibiting the KLK8-PAR1 signaling axis.

## 4. Discussion

Diabetes exacerbates diastolic and systolic heart failure following myocardial infarction, leading to the development of myocardial microvascular complications clinically known as DCM [28]. The prevention and treatment of DCM have received much attention; however, due to the unclear pathogenesis of DCM, effective measures to control diabetes-related cardiovascular complications are lacking [29]. Thus, an animal model of DCM needs to be established to understand the pathogenesis of DCM. Dysregulation of glucose and lipid metabolism associated with diabetes is the primary reason behind the onset of DCM, with long-term control of blood sugar levels serving as a cornerstone of diabetes management [30]. Our findings showed that mice fed a high-sugar diet al.ong with STZ induction presented a considerable increase in FBG levels, impaired glucose tolerance, exacerbated insulin resistance, and abnormal lipid levels, similar changes were reported in the T2DM model described by Xu et al. [31].

Cardiac fibrosis is a ubiquitous structural feature that occurs among individuals suffering from DCM, culminating in heart failure and compromised cardiac performance [32]. Kong et al. hypothesized that irrespective of the initial causative agent, cardiac injury leads to a sustained fibrotic reaction, characterized by high levels of profibrotic growth factors and cytokines in the circulation and myocardium, thereby instigating fibrotic cascades [33]. A poor understanding of fibrogenesis underlies the slow treatment of cardiac fibrosis. This study showed notable pathological alterations in the hearts of DCM-afflicted mice, manifesting as impaired cardiac function, an increase in collagen accumulation, and high expression of fibrotic markers. These findings showed the key role that cardiac fibrosis plays in the progression of DCM in mice administered an HSF along with STZ exposure, thus indicating that the T2DM animal model can serve as a source of novel therapeutic strategies for DCM. Hence, we examined the role of GAS in treating DCM.

Derived from the Chinese herb *G. elata*, GAS is a natural phenolic compound with several pharmacological activities. Drugs currently used primarily in the clinical setting for treating T2DM include metformin, sulfonylureas, thiazolidinediones, insulin, and α-glucosidase inhibitors [34]. However, these drugs have relatively high side effects. Metformin, a small-molecule antihyperglycemic agent mainly used clinically for the treatment of T2DM, was isolated from an extract of *Galega officinalis* [35]. Its success has encouraged the search for other drugs. As an effective antihyperglycemic agent predominantly used for treating T2DM (T2DM), metformin is commonly used as a positive control in experimental T2DM studies [36]. Our findings showed that consistent with the efficacy of clinically used metformin, GAS has some hypoglycemic effects. Wan et al. reported that GAS significantly decreased hepatic lipid accumulation induced by a high-fat diet (HFD) or high-fat high-cholesterol diet (HFHC) in mice [11], a finding confirmed in a DCM model. As described by Pathol and Gourdie, the primary effector cells involved in cardiac fibrosis are CFs, which are stimulated to increase the levels of cardiac fibrogenic growth factors, bind to their receptors, and then trigger the activation of signaling pathways, including the transforming growth factor-β1 (TGFβ) pathway, which plays a key role in the development of cardiac fibrosis. TGFβ1 stimulates fibroblast differentiation and proliferation and increases the synthesis of many extracellular matrix proteins, including collagen [6]. Our in vitro experiments revealed that CFs exposed to high-glucose palmitate exhibit myofibroblast transition, which increases the expression of TGFβ1, a response mitigated by GAS intervention. Cell scratch assays and α-SMA immunofluorescence confirmed these changes. Our findings suggested that GAS therapy ameliorates myocardial fibrosis associated with DCM.

Kallikreins (KLKs), comprising 15 serine proteases (KLK1-KLK15), are a cohort of trypsin and trypsin-like serine proteases distributed across various tissues in the body [37]. Among them, KLK8, a constituent of the KLK family, was found by Du et al. to play a role in dilated cardiomyopathy (DCM), which is typified by cardiac fibrosis. The absence of KLK8 effectively ameliorated diabetes-induced cardiac fibrosis. Silencing KLK8 significantly mitigated high-glucose-induced endothelial-mesenchymal transition, whereas overexpressing KLK8 in endothelial cells promoted endothelial-mesenchymal transition [13]. We found that upregulating KLK8 increases the fibrotic response of CFs and KLK8 siRNA can considerably alleviate the fibrotic alterations in CFs exposed to high glucose levels. Many studies have revealed a correlation between an increase in the levels of KLK8 and the pathogenesis of various diseases, such as pancreatic cancer [16] and depression [38]. The key function of KLK8 in a broad spectrum of ailments has facilitated the advancement and evaluation of targeted therapy for KLK8. An array of drug modalities, including peptide-based and small-molecule agents, monoclonal antibodies, engineered natural molecules, noncovalent inhibitors, and antibodies, are used in the development of KLK8-targeted therapeutic strategies [13, 14]. In this study, we found that GAS can hinder the upregulation of KLK8, impeding the differentiation, collagen synthesis, and migration of CFs subjected to HG-PA, as confirmed by α-SMA immunofluorescence. However, further studies need to be performed to elucidate the intricate molecular mechanisms through which GAS inhibits the expression and activation of KLK8.

Hua et al. revealed that KLK8 promotes the proliferation and migration of colorectal cancer cells by activating PAR1 [17]. Cao et al. found that inhibiting the protease-activated receptor PAR1 or PAR2 pathways significantly reduces hypertrophy in cardiomyocytes exposed to KLK8 [14]. PARs can mediate cellular harm and signaling in T2DM [39]. PAR1 activation can promote proliferation, phenotypic transformation, and the synthesis of extracellular matrix proteins in CFs [40]. Our experimental findings revealed that the fibrotic activation of CFs exposed to OE-KLK8 can be reversed by the PAR1 antagonist SCH79797 but not the PAR2 antagonist FSLLRY-NH2. Similarly, consistent with the potential therapeutic target of PAR1 reported by Chandrabalan A as a prophylactic measure against cardiovascular diseases, we found that PAR1 acts as a key regulator of platelet function, whereas PAR2 promotes inflammatory responses [41]. Based on these findings, we proposed that GAS may counteract HG-PA-induced cardiac fibrosis by engaging in the KLK8/PAR1 signaling axis in CFs. Our data indicated that PAR1 antagonists impede fibrotic responses in CFs exposed to HG-PA. We found that GAS regulates PAR1 activity to mitigate the progression of fibrosis in CFs exposed to OE-KLK8. These results suggested that GAS is involved in the HG-PA-induced differentiation, collagen synthesis, and migration of CFs through the inhibition of the KLK8-PAR1 signaling axis.

A recent study revealed that the increase in KLK8 expression in diabetic cardiac fibrosis may be associated with an increase in TGF-β/Smad signaling and profibrotic transcription factors [13]. The activation of PAR-1 facilitates ERK1/2 MAPK signaling, which in turn regulates the effect of TGF-β on extracellular matrix (ECM) protein synthesis. Moreover, activation of the ERK1/2 MAPK pathway increases the expression of PAR-1 under pathological conditions, stimulating the release of TGF-β and subsequent Smad3 signaling [42]. Our findings confirmed that blocking PAR-1 activity with GAS can mitigate the increase in KLK8-induced TGF-β and Smad3 signal generation. These findings suggested that tetramethylpyrazine may impede the production of TGF-β and Smad3 signaling by inhibiting the KLK8-PAR1 signaling axis. Further investigation is needed to assess the role of these signaling pathways in driving cardiac fibroblast (CF) differentiation, collagen synthesis, and cell migration through the KLK8-PAR1 signaling axis in CFs.

Our findings suggested that in murine models relevant to clinical diabetes, GAS can manage the onset and progression of DCM. Targeting cardiac fibrosis is an important and effective strategy to treat diabetes. KLK8 may serve as a novel mediator for targeting PAR1 receptor-induced and HG-PA-induced cardiac fibrosis. The application of GAS to decrease the activation of the KLK8-PAR1 signaling axis triggered by HG-PA is an innovative therapeutic approach to prevent the development and progression of DCM marked by cardiac fibrosis. However, in this study, we did not validate the precise effect of GAS on the KLK8-PAR1 signaling axis in vivo. Although the effect of the GAS component on organ function and histological attributes in mice is minimal [11], further investigation is necessary to establish its long-term safety and toxicological profile to confirm its appropriateness for the clinical management of DCM.

In summary, it is the first time that our results indicate GAS ameliorating DCM both in vivo and in vitro, suggesting GAS is a beneficial potentially promising candidate for DCM in clinic, which might be offer alternative options for DCM in the future. The mechanism of GAS is directly targeted KLK8 to inhibit the KLK8-PAR1 signaling axis, which may be presented the potentially novel DCM pathogenesis and potential drug interventions. Moreover, future clinical trials of GAS therapy for DCM should be extensively designed to evaluate the therapeutic mechanisms and improve clinical diagnostic and treatment strategies.

## 5. Conclusions

To summarize, our results suggested that GAS inhibits DCM, which is characterized by cardiac fibrosis, most probably by inhibiting the KLK8-PAR1 signaling axis, and thus, the process of DCM.

## Supplementary Information

Supplementary Material 1.

## Data Availability

The data in this study are available from the corresponding author upon reasonable request.
