# Supplementary material for: Gastrodin attenuates diabetic cardiomyopathy characterized by myocardial fibrosis by inhibiting the KLK8-PAR1 signaling axis
Source: Chin Med. 2024 Nov 22;19:164. doi: 10.1186/s13020-024-01035-4 (PMC11583739; doi:10.1186/s13020-024-01035-4)
Supplement: Supplementary file 1 — Supplementary Material 1. [file 13020_2024_1035_MOESM1_ESM.pdf]

Sept 22, 2024

Certificate no.: AYD2024091807
Total words edited: 7647

CERTIFICATE OF EDITING

This is to certify that the manuscript attached herewith was edited for the proper English language, grammar, punctuation, spelling, and overall style by me or under my supervision.

Manuscript Title

Gastrodin attenuates diabetic cardiomyopathy characterized by myocardial fibrosis by inhibiting the KLK8-PAR1 signaling axis

Feroz Khan, MS, PhD,
Ex. Faculty: University of California (UCSF), San Francisco, CA
Email: service@aiyidesci.com
www.aiyidesci.com

Disclaimer: The authors received an editable copy of the text and were free to alter/edit/reject/accept changes after editing. Hence, the version under consideration for publication may be different from the one we have provided; in such a case, the text attached herewith should be considered authentic or unaltered. Besides, many times, authors get selective editing that excludes headings, such as materials, methods, tables, figure legends, and/or bibliography. Even, in this case, only the text attached herewith should be considered edited.
